# Evaluation of cytotoxicity and bleaching efficacy of gels with calcium polyphosphate and violet LED

**DOI:** 10.1590/0103-644020256586

**Published:** 2026-01-30

**Authors:** Larissa de Jesus Gomes, Rafael Antonio de Oliveira Ribeiro, Mariangela Ivette Guanipa Ortiz, Klaus Rischka, Carlos Alberto de Souza Costa, Débora Alves Nunes Leite Lima

**Affiliations:** 1 Department of Restorative Dentistry, Piracicaba School of Dentistry, State University of Campinas - UNICAMP Av. Limeira, 901 - Areião, Piracicaba - SP, 13414-903 Brazil; 2 Department of Dental Materials and Prosthodontics, School of Dentistry, São Paulo State University - UNESP, Rua Humaita, Araraquara - SP 14801‑903, Brazil; 3 Fraunhofer Institute for Manufacturing Technology and Advanced Materials IFAM, Wiener Str 12,28359 Bremen, Germany; 4 Department of Physiology and Pathology, Araraquara School of Dentistry, Sao Paulo State University - UNESP, Rua Humaita, Araraquara - SP 14801‑903, Brazil

**Keywords:** Tooth Bleaching, hydrogen peroxide, cytotoxicity, polyphosphates, light

## Abstract

To evaluate the whitening effect and cytotoxicity of low-concentration hydrogen peroxide (HP 10%) combined with calcium polyphosphate (CaPP) submicroparticles, irradiated or not with a violet LED (LEDv). Bovine tooth samples were divided into 6 groups (n=6): Control Group (no treatment); LED; Manipulated 10% HP (HP10%); Manipulated 10% HP + CaPP 1.5% w/t (HP10%_CaPP); Manipulated 10% HP + LED (HP10%+L); and Manipulated 10% HP + CaPP 1.5% w/t + LED (HP 10%_CaPP+L). The treatments were applied to the enamel of disks mounted in artificial pulp chambers. The whitening effect (ΔE00 and ΔWID) was measured, and the extracts were applied to MDPC-23 cells, assessing cell viability, oxidative stress, and HP diffusion. The data were analyzed using generalized linear models, and the Kruskal-Wallis/Dunn tests were applied for WID (a=5%). Cell viability was significantly reduced in all HP groups compared to controls (Control: 100.15 ± 1.71%; LED: 99.89 ± 2.43%), with the highest viability in HP10%_CaPP+L (72.17 ± 4.14%) and the lowest in HP10% (54.48 ± 1.72%). Oxidative stress was lowest in HP10%_CaPP+L (2.91 ± 0.17) and highest in HP10%+L (3.77 ± 0.15). HP diffusion was markedly reduced with CaPP (HP10%_CaPP+L: 2.93 ± 0.18 vs. HP10%: 4.24 ± 0.10). In terms of color analysis, the addition of CaPP did not alter the bleaching efficacy. The addition of CaPP to hydrogen peroxide, combined with violet LED, demonstrated effective bleaching action, positive indicators of cytocompatibility, increased cell viability, and reduced oxidative stress

## Resumo

Avaliar o efeito clareador e a citotoxicidade do peróxido de hidrogênio de baixa concentração (PH 10%) combinado com submicropartículas de polifosfato de cálcio (PPCa), irradiado ou não com um LED violeta (LEDv). As amostras de dentes bovinos foram divididas em 6 grupos (n=6): Grupo controle (sem tratamento); LED; Manipulado com 10% de PH (PH10%); Manipulado com 10% de PH + PPCa 1,5% p/t (HP10%_CaPP); Manipulado com 10% de PH + LED (PH10% + L); e Manipulado com 10% de PH + PPCa 1,5% p/t + LED (HP 10%_CaPP+L). Os tratamentos foram aplicados no esmalte dos discos montados em câmaras pulpares artificiais contendo células MDPC-23. O efeito de clareamento (ΔE00 e ΔWID) foi avaliado, e os extratos obtidos foram usados para avaliação da viabilidade celular, do estresse oxidativo e da difusão do pH. Os dados foram analisados por meio de modelos lineares generalizados, e os testes de Kruskal-Wallis e Dunn foram aplicados para WID (α = 5%). A viabilidade celular foi significativamente reduzida em todos os grupos com PH quando comparados aos controles (Controle: 100,15 ± 1,71%; LED: 99,89 ± 2,43%), sendo mais alta em PH10%_PPCa+L (72,17 ± 4,14%) e mais baixa em PH10% (54,48 ± 1,72%). O estresse oxidativo foi menor em PH10%_PPCa+L (2,91 ± 0,17) e maior em PH10%+L (3,77 ± 0,15). A difusão de PH também foi reduzida pela adição de PPCa (PH10%_PPCa+L: 2,93 ± 0,18 vs. PH10%: 4,24 ± 0,10). Em relação à viabilidade celular, ao estresse oxidativo e à difusão de pH, a adição de PPCa apresentou melhores resultados, com a melhor combinação encontrada no grupo em que o PPCa foi combinado com LED. Em termos de análise de cor, a adição de PPCa não alterou a eficácia do clareamento. A adição de PPCa ao peróxido de hidrogênio, combinada com LED violeta, demonstrou ação clareadora eficaz, indicadores positivos de citocompatibilidade, aumento da viabilidade celular, redução do estresse oxidativo e menor perfusão de peróxido de hidrogênio nas células pulpares.

**Figure:**
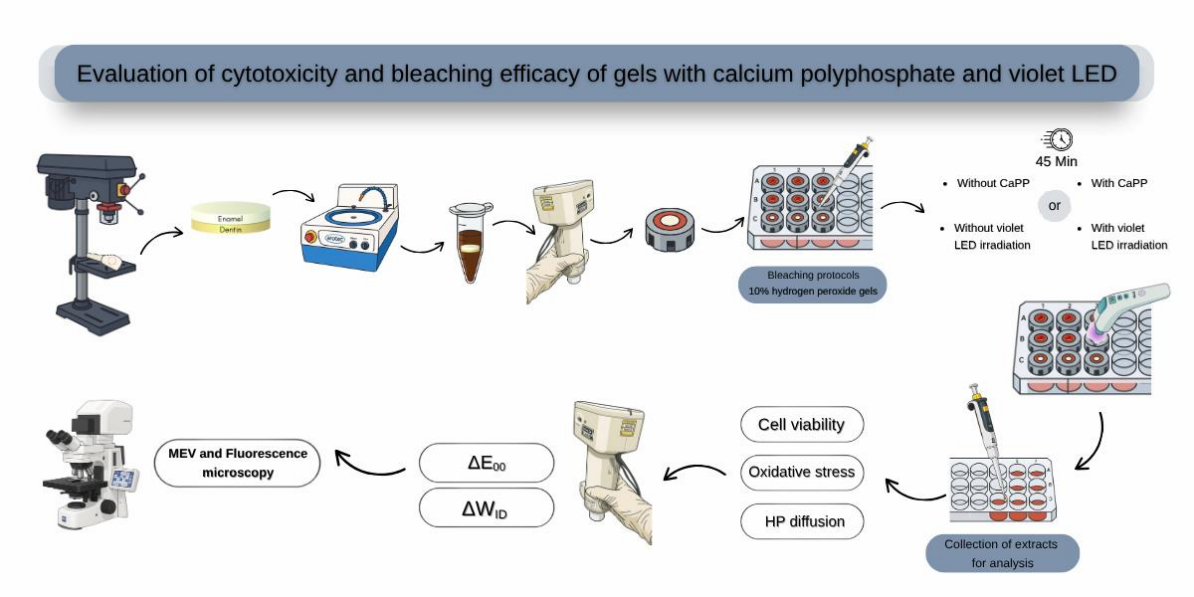

## Introduction

There are various formulations of substances available for vital tooth bleaching, with the most commonly used being carbamide peroxide and hydrogen peroxide (HP) gels, whose concentrations vary according to the instituted treatment modality^1^. For in-office bleaching, higher concentrations of hydrogen peroxide (35-40%) are commonly employed, whereas lower concentrations (4-15%) are typically recommended for at-home, dentist-supervised bleaching procedures. Additionally, the combination of bleaching gels with various light sources has been studied to enhance the chemical decomposition of HP, thereby increasing its effect and reducing application time^1^ ^,^ ^2^.

Among the various light sources investigated to enhance HP degradation, violet LED has been studied due to its specific wavelength, which gives it the ability to accelerate HP degradation. Some studies have suggested that violet light may contribute to improved whitening results^3^ ^,^ ^4^ ^,^ ^5^. The bleaching action of peroxides is based on their ability to trigger a chemical oxidation reaction, generating free oxygen molecules^1^. These molecules, due to their low molecular weight, can penetrate the enamel and dentin, degrading chromogenic agents that cause stains into smaller particles, thus facilitating their removal^6^. However, due to the action of released free radicals, dental bleaching can cause adverse effects^7^, such as alterations in the enamel surface, increased surface roughness, and cytotoxicity. These effects result in symptoms like post-bleaching sensitivity, frequently reported in current bleaching therapies^6^ ^,^ ^7^ ^,^ ^8^. Therefore, it is necessary to develop bleaching gel formulations that reduce or eliminate these side effects^8^.

Regarding hypersensitivity caused by dental bleaching, the low molecular weight of peroxides allows their diffusion through the enamel and dentin, potentially increasing their cytotoxic potential and ability to damage the dental pulp^10^ through inflammatory reactions that can lead to pulpitis or even pulp necrosis. These effects are related to the ability of HP and its by-products to penetrate the cell membrane and release free radicals, generating oxidative stress and facilitating lipid peroxidation, resulting in decreased cell viability and changes in cell morphology^11^. Furthermore, bleaching gels promote the release of pro-inflammatory cytokines, which ultimately hinder cellular regeneration^10^ ^,^ ^12^. Therefore, it is crucial to conduct new studies to develop alternative procedures with lower cytotoxicity to avoid damage such as pulpitis or pulp necrosis^11^.

The need to develop bleaching formulations that maintain bleaching efficacy while reducing the adverse effects of this treatment remains. Bioactive materials have emerged as an excellent alternative to traditional bleaching treatments, as they can interact directly with cells by mimicking biological substances^13^ ^,^ ^14^ or even promote chemical reactions that allow the precipitation of calcium phosphates when in contact with living tissues. In this regard, bioactive glasses and calcium polyphosphate (CaPP) stand out^15^ ^,^ ^16^. Despite being recently discovered, CaPP presents itself as a promising particle as it is responsible for the mineralization of living tissues, explained by the binding of calcium to the mineral matrix of hydroxyapatite^15^ ^,^ ^17^. Thus, it can reduce the undesirable effects of bleaching treatment through its ability to release calcium and phosphate ions that favor the remineralization process, filling surface defects and preventing or mitigating the adverse effects of bleaching treatment^16^.

Considering the importance of making the tooth whitening process safer and the development of new formulas that utilize bioactive elements, this study aimed to investigate the effect of a low-concentration HP-based whitening gel (10%) with the addition of sub-microparticles of CaPP, with and without the association of violet LED, evaluating its trans-enamel and trans-dentin cytotoxicity, whitening efficacy, and the kinetics of HP degradation. The hypotheses tested were as follows: 1. The cytotoxic potential and hydrogen peroxide diffusion of whitening agents containing CaPP will differ from those of whitening agents without CaPP when used in conjunction with a violet LED light source, and 2. The efficacy of whitening agents containing CaPP will be different from that of whitening agents without CaPP when used with a violet LED light source.

## Materials and methods

### Sample preparation

A total of 36 standardized specimens (n=6) with a diameter of 5.6 mm, obtained from bovine incisors, were prepared using a bench drill (FSB 16 Pratika, SchμLtz, Joinville, SC, Brazil) with a diamond trephine drill (Dinser Diamond Drills Ltda., São Paulo, SP, Brazil), based on sample calculation. After obtaining the discs, 400 and 600-grit water sandpapers (T469-SF-Noton, Saint-Gobain Abrasivos Ltda., Jundiaí, SP, Brazil) were used to smooth the dentin surface. For the enamel, smoothing and polishing were done with 600, 1200, 2500, and 4000 grit sandpapers and 1 and 1/4 felt discs to standardize their thickness^17^, similar to human lower incisors, i.e., 2.3 mm (1 mm of enamel and 1.3 mm of dentin)^10^.

### Standardization of the initial disc color

After standardizing the thicknesses, the specimens were subjected to staining to standardize the initial color of the teeth across all groups^8^. Initially, to remove the smear layer, EDTA at 0.5 M was applied to the dentin surface for 30 seconds to remove the smear layer^18^. A black tea solution (Leão Jr. S.A., Fazenda Rio Grande, PR, Brazil) was prepared by steeping 3.2 g of tea in 100 mL of boiling water for 5 minutes. The discs were then immersed in the black tea solution for 24 hours. After this period, enamel prophylaxis was performed using a pumice stone (S.S. White, Rio de Janeiro, RJ, Brazil) and water to remove any superficial pigments not absorbed. The specimens were then kept in distilled water for 24 hours to facilitate the removal of pigments not fully absorbed by the dentin^8^; dentin isolation was not performed to avoid interference with hydrogen peroxide diffusion through the varnish film formed.

### Application of the bleaching protocol

Table 1 outlines the treatment groups used in the study. Each bleached group underwent a session with a standardized total duration of 45 minutes, employing a whitening gel with a 10% concentration of hydrogen peroxide (HP). The gels (20 µL) were applied to the enamel surface of the teeth, and some groups were exposed to a violet LED light source (Bright Maxx Whitening HPotoclearer, MMOptics Ltda, São Carlos, SP, Brazil) with a wavelength between 405 and 410 nm. The teeth received 60-second irradiation cycles, followed by 30-second intervals throughout the entire 45-minute treatment period. After the bleaching process was completed, the gel was removed from the tooth surface through rinsing with distilled water. The control group did not undergo any treatment. After the bleaching process was completed, the gel was removed from the tooth surface through rinsing with distilled water^19^.

**Table 1:** Groups according to the instituted treatment (n = 6)

Group	Treatment instituted	Time	Composition
Control	No treatment	0 min	-
LED	LED irradiation	20 min	-
HP10%	Application of the bleaching gel	45 min	Hydrogen peroxide, glycerin, distilled water, Carbopol 940, sodium hydroxide, propylene glycol, sodium polyphosphate
HP10%_CaPP	Application of the bleaching gel + CaPP	45 min	Hydrogen peroxide, glycerin, calcium polyphosphate particles, distilled water, Carbopol 940, sodium hydroxide, propylene glycol, sodium polyphosphate
HP10% + L	Application of the bleaching gel + LED irradiation	45 min	Hydrogen peroxide, glycerin, distilled water, Carbopol 940, sodium hydroxide, propylene glycol, sodium polyphosphate
HP 10%_CaPP+L	Application of the bleaching gel + CaPP + LED irradiation	45 min	Hydrogen peroxide, glycerin, calcium polyphosphate particles, distilled water, Carbopol 940, sodium hydroxide, propylene glycol, sodium polyphosphate

### Manipulation of bleaching gels

The whitening gels were prepared in a dark room, where their components were carefully weighed using a precision balance to ensure standardization across the different preparations. Two solutions were prepared: one containing hydrogen peroxide (HP) and another with the thickening agent. For the groups that received treatment with CaPP, this compound was added to the thickening agent solution. Initially, the CaPP was diluted in distilled water and then heated in a water bath at 50°C for 15 minutes. After that, it was combined with the other reagents, and the mixture was homogenized using a mixer at a speed of 2,000 to 2,500 rpm for 10 minutes. All solutions were stored in Eppendorf tubes under refrigeration (between 1-8°C) and kept away from light to prevent peroxide degradation. The solutions were brought to room temperature 30 minutes before use, and at the time of application, the two solutions were mixed^16^.

Two solutions were used to prepare the whitening gel, as in commercial gels, one being a peroxide solution and the other a thickener, as described below: Solution 1: Glycerol - Glycerin PA-ACS - C3H8O3 (Êxodo Científica); 1,2-Propanediol - PA-ACS - C3H8O2 (Synth); Carbopol 940 - (C3H4O2)n PA (ACS científica); Citric acid - PA-ACS - HCl (Sigma); sodium hydroxide - NaOH - PA (Dinâmica); hydrogen peroxide - H2O2 PA (Dinâmica). Solution 2: H2O; Glycerol - Glycerin PA-ACS - C3H8O3 (Êxodo Científica); 1,2-Propanediol - PA-ACS - C3H8O2 (Synth); Carbopol 940 - (C3H4O2)n PA (ACS científica); Sodium polyphosphate NaPP - P.A - (NaPO3)N (sigma); Calcium polyphosphate - CaPP (Particle handled by the group); sodium hydroxide - NaOH - PA (Dinâmica)^16^. 

Solution 1 was mixed (600 rpm) in a plastic device protected from light, in a cold environment, for 75 min. Solution 2 was mixed in a plastic device. After adding all the components, it was put into a Speed Mixer (1800 rpm / 1.5 min) and then into a shaker table at 250 rpm/90 min or until a homogenized solution was obtained^16^. 

The thickener used was Carbopol 940, with the aim of increasing the viscosity of the product after mixing solution 1 with solution 2, with a ratio of 3:1, as in commercial gels. The concentration was measured immediately after mixing with a concentration of 10.1% hydrogen peroxide. With regard to pH, pH values were measured at 15, 30, and 45 min and ranged from 5.5 to 6, which characterizes a slightly acidic pH and contributes to the stability of the peroxide^16^.

CaPPs were synthesized and characterized following the coprecipitation method for later incorporation into an experimental bleaching gel. SEM analysis showed that the particles had a spherical morphology. The presence of Ca and P was detected using the EDX system. The particles presented a Ca:P atomic ratio of 1.11. The CaPP particles showed a sub-microparticle size (135.7 ± 80.95 nm). The hydrodynamic radius of the CaPPs showed a distribution of 257.1 nm (± 26.52 nm), with a polydispersity index of 0.43 (±0.05) and a correlation of 0.90. The CaPP concentration used was 1.5% CaPP; this concentration was chosen based on previous studies conducted by our research group, which found that this concentration allows for better use of PPCa, yielding better results^16^.

### Evaluation of the cytotoxicity level of the bleaching gel

### MDPC-23 odontoblastic cell culture

Isolated cells from the odontoblastic lineage, MDPC-23, were cultured in cell culture plates (KASVI Imp, São José dos Pinhais, PR, Brazil) 24 hours before the whitening treatment. The cells were maintained in Dulbecco's Modified Eagle's Medium (DMEM; GIBCO, Grand Island, NY, USA) enriched with 10% fetal bovine serum (FBS; GIBCO), 100 IU/mL of penicillin, 100 µg/mL of streptomycin, and 2 mmol/L of glutamine (GIBCO) in a humidified atmosphere at 37°C with 5% CO2 and 95% air.

### Experimental procedure

The specimens were placed in artificial pulp chambers (APCs), using silicone rings sealed with utility wax (Technew, Rio de Janeiro, RJ, Brazil) to prevent the leakage of the whitening gel into the area corresponding to the pulp chamber^9^. After assembly, the setups were sterilized with ethylene oxide (Acecil, Central de Esterilização Comércio e Indústria Ltda., Campinas, SP, Brazil). The APCs were then individually placed in sterile 24-well plates (KASVI Imp.) containing 1 mL of culture medium, which remained in direct contact with the dentin while the enamel was exposed during the whitening sessions. After the first whitening session, the content of the wells (extract) was collected for further analysis.

### Cell Viability Assessment (MTT Assay)

To assess cell viability, the MTT assay was conducted using (3-(4,5-dimethylthiazol-2-yl)-2,5-diphenyltetrazolium bromide; Sigma-Aldrich; n=6)^9^. The collected extract was applied to MDPC-23 cells, and after 1 hour, it was aspirated. The cells that remained adhered to the bottom of the plate were then incubated with a solution composed of 90 μL DMEM and 10 μL MTT solution. The MTT solution was prepared at a concentration of 5 mg/mL in phosphate-buffered saline (PBS; GIBCO).

The samples were incubated for 4 hours, during which the enzyme succinate dehydrogenase in viable cells produced blue formazan crystals. These crystals were dissolved in 100 μL isopropanol (Sigma-Aldrich), acidified with 0.04 N HCl (Sigma-Aldrich), and the absorbance was measured at 570 nm (Synergy H1, Biotek, Winooski, Vermont, USA). The control group, which did not receive any whitening treatment, was considered to have 100% cell viability and served as a reference to calculate the percentage of cell viability in the experimental groups.

### Oxidative Stress Analysis

To evaluate oxidative stress, the production of reactive oxygen species (ROS) by the cells was measured immediately after whitening^9^. Prior to exposure to the extracts, MDPC-23 cells were incubated for 30 minutes with a carboxy-H2DCFDA probe (Invitrogen, San Francisco, CA, USA) at a concentration of 10 μg/mL. After exposure to the extracts, the fluorescence intensity of the cells was measured with excitation at 592 nm and emission at 517 nm (Synergy H1). The control group values were used to normalize the data, allowing for the quantification of the increase in fluorescence intensity in each experimental group.

### Quantification of HP in Extracts

To stabilize the pH, 100 μL of the extracts were added to tubes containing 900 μL of acetate buffer (2 mol/L, pH 4.5) (5 mL test tubes, KASVI Imp.). Next, 500 μL of this solution was transferred to tubes containing water and violet leucocrystal violet dye (0.5 mg/mL; Sigma-Aldrich). After mixing, 50 μL of a 1 mg/mL radish peroxidase solution (Sigma-Aldrich) was added. The absorbance of the solutions was measured using a spectrophotometer at a wavelength of 596 nm. A standard curve of known quantities of HP was used to convert the optical density values obtained from the samples into μg of HP per mL of extract^9^.

### Color measurement

Color measurements of the specimens were performed using a portable spectrophotometer (Reflection UV-Visible Spectrophotometer, Color Guide, BYK Gardner GmbH, Geretsried, Germany), positioned on the discs with the aid of a standardized support to ensure reproducibility. Color values were obtained according to the CIE L*a*b* system at two experimental time points: after staining (T1) and 72 h after bleaching (T2).

Prior to each color measurement, the discs were stored for 72 h in a container with the dentin in contact with cotton soaked in deionized water and the enamel in contact with cotton soaked in a saliva-like solution (containing 3.9% monobasic potassium phosphate, 3.6% calcium chloride, 2% sodium chloride, 2% potassium chloride, 3.7% magnesium chloride, 0.2% phenochem, 10% natrosol gel, and distilled water q.s.p.) to standardize the hydration level.

After the staining protocol, the spectrophotometer was positioned on the specimens using a standardized support, allowing the L*a*b* values to be obtained. Discs with statistically similar L* values were selected and allocated among the groups to ensure comparability. The specimens were placed in a silicone matrix to expose the enamel surface, and a new measurement was performed after the bleaching protocol using the exact spectrophotometer operated by the same examiner. The L*a*b* values obtained after staining and 72 h after bleaching were used to calculate the color change (ΔE00) and the whitening index (ΔWID)^21^ according to the following equations:

$$\Delta E_{00}=\;\sqrt{\left[{\left(\frac{\Delta L^{'}}{K_{L}S_{L}}\right)}^{2}+\;{\left(\frac{\Delta C^{'}}{K_{C}S_{C}}\right)}^{2}+\;{\left(\frac{\Delta H^{'}}{K_{H}S_{H}}\right)}^{2}+\;R_{T}\left(\frac{\Delta C^{'}}{K_{C}S_{C}}\right)\left(\frac{\Delta H^{'}}{K_{H}S_{H}}\right)\right]}\;\;\;$$

$$WID\;=\;{0.511L}^{*}\;-\;{2.3424a}^{*}\;-\;{1.100b}^{*}$$

ΔWI_D_ = WID*posttreatment* - WI*baseline*, where ΔL' corresponds to the brightness value difference, ΔC' represents the chroma difference, and ΔH' is the hue difference based on the CIEDE2000 metric. The parameters to adjust the coordinate values in a function corresponding to color variations are represented by SL, SC, and SH. Meanwhile, KL, KC, and KH are correction parameters for the experimental conditions, and RT is a parameter that considers the interaction of differences between chroma and hue in the blue region^21^ ^,^ ^22^. Next, the color parameters ΔE_00_ and ΔWI_D_ obtained for the control and experimental groups were subjected to statistical analysis.

### Statistical Analysis

Initially, descriptive and exploratory data analyses were performed, with variables described by means, standard deviations, medians, minimum, and maximum values for each group. These preliminary analyses indicated that the data did not meet the assumptions for a traditional analysis of variance with a general linear model. Therefore, generalized linear models were used to evaluate the effect of the groups on the variables of percentage of cell viability, oxidative stress, H_2_O_2_ diffusion, and Δ E_00_. For the WID data, which did not fit a known distribution, non-parametric Kruskal-Wallis and Dunn tests were applied. All analyses were conducted using R software, with a significance level of 5%.

### Results

**Figure 1:**
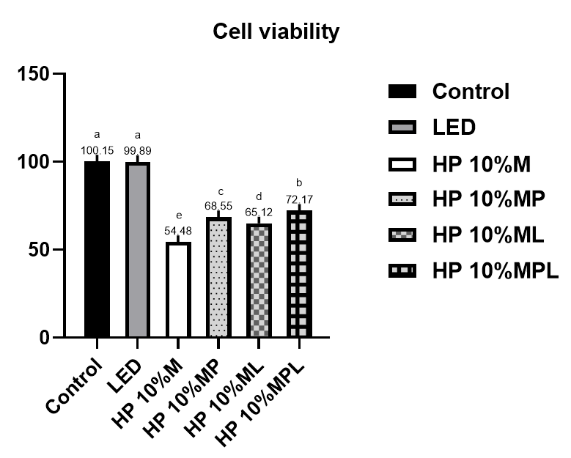
Cell viability (%) according to the group. p<0.0001. Different letters indicate statistically significant differences between groups (p≤0.05).

The results shown in Figure 1 indicate that Cell viability was significantly lower in all experimental groups compared to the controls (Control: 100.15 ± 1.71%; LED: 99.89 ± 2.43%) (p<0.05). Among the experimental groups, the highest viability was observed in the HP 10%_CaPP+L (72.17 ± 4.14%), followed by HP10%_CaPP (68.55 ± 5.09%) and HP10% + L (65.12 ± 3.36%). The lowest values were seen in HP10% (54.48 ± 1.72%) (p<0.0001).

**Figure 2:**
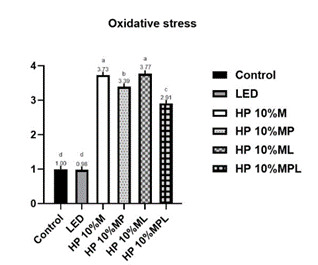
Oxidative stress (fluorescence intensity) according to the group. p<0.0001. Different letters indicate statistically significant differences between groups (p≤0.05).

Regarding oxidative stress, as described in Figure 2, the control (1.00 ± 0.02) and LED (0.98 ± 0.05) groups showed significantly lower values than the groups compared to the HP groups (p<0.05). Among the HP groups, HP 10%_CaPP+L showed the lowest oxidative stress (2.91 ± 0.17), followed by HP 10%_CaPP (3.39 ± 0.12). The highest oxidative stress was observed in HP10% (3.73 ± 0.16) and HP10% + L (3.77 ± 0.15), with no statistical difference between them (p<0,0001). These findings indicate a protective effect of CaPP.

**Figure 3:**
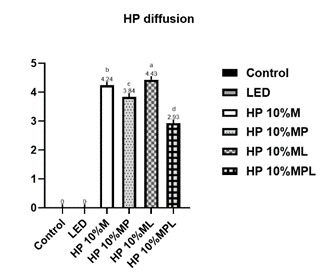
HP diffusion (fluorescence intensity) according to the group. p<0.0001. Different letters indicate statistically significant differences between groups (p≤0.05).

Figure 3 presents the results related to HP diffusion. For this analysis, the control and LED groups were not considered because they did not contain HP. The group with the least diffusion was HP 10%_CaPP+L (2.93 ± 0.18), followed by HP 10%_CaPP (3.84 ± 0.18). The highest diffusion results were in the HP10% group (4.24 ± 0.10), followed by HP10% + L (4.43 ± 0.12), with a statistical difference between them (p<0.0001). These results suggest that CaPP effectively reduced HP penetration through enamel and dentin.

**Figure 4:**
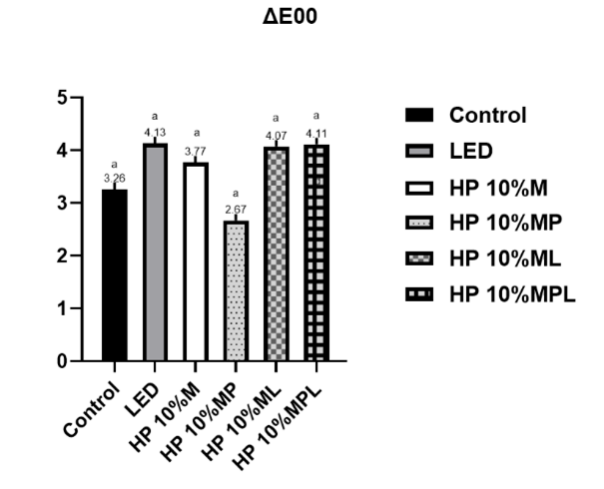
ΔE_00_ according to the group. p=0.0094. Different letters indicate statistically significant differences between groups (p≤0.05).

In terms of color variation measured by ΔE_00_, as seen in Figure 4, the HP 10%_CaPP+L (4.11 ± 0.55) group showed the most tremendous color variation, but there was no statistically significant difference between the groups (p=0.1170).

**Figure 5:**
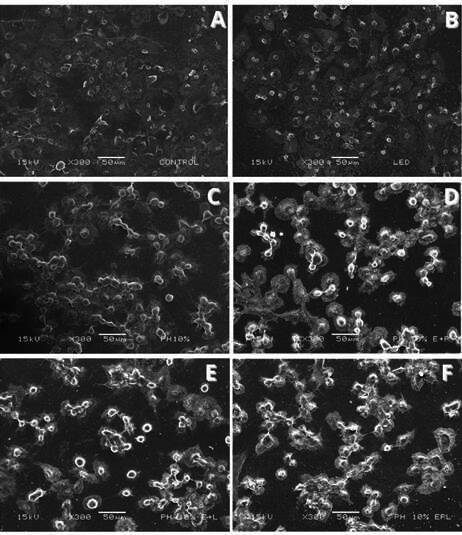
WID according to the group. p=0.0120. Different letters indicate statistically significant differences between groups (p≤0.05).

Regarding the ΔWID results, the control (-0.88 ± 4.63) and LED groups (2.68 ± 6.60) showed the lowest indices. In contrast, the groups that received HP had higher values, with no statistical difference between the groups (p = 0.0120), as described in Figure 5.

**Figure 6:**
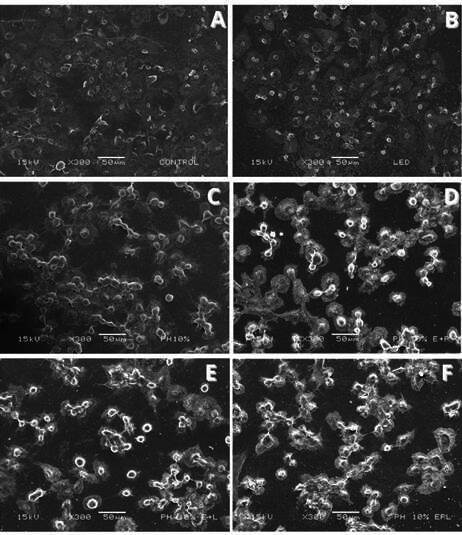
Scanning electron microscopy (SEM) images showing cell morphology after different bleaching treatments. (A) Control: cells with preserved morphology and uniform surface; (B) LED: cells with morphology similar to the control, without relevant alterations; (C) HP10%: cells with morphological changes; (D) HP10%_CaPP: presence of cells with more preserved morphology compared to HP alone; (E) HP10% + L: greater irregularity in cell morphology; (F) HP 10%_CaPP+L: cells with morphology closer to the control, suggesting a protective effect of CaPP associated with LED.

**Figure 7:**
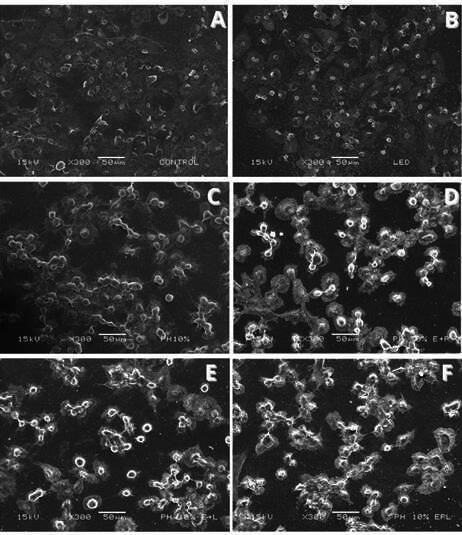
Fluorescence microscopy (Live/Dead assay) showing cell viability after different bleaching treatments. Live cells are shown in green and dead cells in red. (A) Control: high density of viable cells; (B) LED: similar pattern to control; (C) HP10% : higher proportion of dead cells, evidenced by increased red fluorescence; (D) HP10%_CaPP: greater number of viable cells compared to HP alone; (E) HP10% + LED: increased cell mortality; (F) HP 10%_CaPP+L : improved cell viability compared to HP 10% alone, suggesting a protective effect of CaPP.

In Figures 6 and 7, images from SEM and fluorescence microscopy are shown according to groups. Allowing for the observation of cell morphology through SEM and the comparison of healthy (green) to dead (red) cells in fluorescence microscopy. SEM images (Figure 6) revealed preserved morphology in the control and LED groups, while HP10% induced apparent alterations. The addition of CaPP (HP10%_CaPP and HP10%_CaPP+L) maintained cell morphology closer to control, whereas HP10%+L showed more irregularity. Fluorescence microscopy (Figure 7) confirmed these findings, with a higher density of viable cells (green) in CaPP-treated groups compared to HP alone, which showed a predominance of dead cells (red).

The groups treated with HP in combination with CaPP and/or LED showed a higher proportion of cells with intact morphology, whereas the group treated solely with HP 10% demonstrated a higher number of dead cells.

## Discussion

Hypothesis 1 was accepted because the cytotoxic potential and HP diffusion of the whitening agents with the addition of CaPP differed from the whitening agents without the addition of CaPP when used in combination with a violet LED light source. The addition of CaPP to the whitening gel reduced trans-enamel-dentin cytotoxicity and HP diffusion. Hypothesis 2 was rejected, as the whitening efficacy of the agents with CaPP did not differ from that of the whitening agents without CaPP when used with a violet LED light source.

Among the groups that received whitening treatment, the groups with the highest viability were HP 10%_CaPP+L and HP 10%_CaPP. In other words, the groups with the addition of CaPP showed positive results. It is important to note that both the LED application and the addition of CaPP, either individually or combined, contributed to improved cell viability (Figure 1). This effect can be attributed to the deposition of inorganic material in the dentinal tubules, which decreases the permeability of HP through these tubules, thereby reducing the amount of this substance reaching the cells^23^. This suggests that the combination of CaPP, a bioinorganic polymer with biomineralization potential, and LED enhances cell viability by reducing mineral loss during whitening.

In the oxidative stress test, the groups without whitening gel (control and LED) showed lower oxidative stress. The groups with the addition of CaPP had better results, presenting lower levels of oxidative stress. On the other hand, HP10% and HP10% + L exhibited the highest oxidative stress (Figure 2), due to the larger diameters of the dentinal tubules resulting from HP-induced demineralization, without the protective inorganic deposition of CaPP. This indicates that CaPP leads to lower oxidative stress.

In the HP diffusion test, only the groups that received HP were considered. Similar to the cell viability test, the addition of CaPP provided better results. The groups with the highest diffusion were those that received gel without the addition of CaPP, regardless of LED application, namely HP10% and HP10% + L (Figure 3). Conversely, the groups that received HP with CaPP showed a lower rate of HP diffusion, indicating that less peroxide reached the studied cells, likely due to the potential remineralization of the tissue previously demineralized by whitening. Thus, CaPP demonstrated promising results by reducing oxidative stress levels and HP diffusion while increasing cell viability in the groups where it was applied. These aspects may reduce the adverse effects of tooth whitening, especially dentin hypersensitivity, in a clinical context ^20^ ^,^ ^23^.

Regarding the color analysis, ΔWID is the parameter that establishes the effectiveness of the bleaching treatment (Figure 5). Therefore, the higher the value, the better the bleaching efficacy. The groups that showed the most significant color change were those treated with HP. Meanwhile, the control and LED groups had a lower bleaching index, corroborating the studies by Gallinari et al.^20^ and Kury et al.^24^. These findings indicated that using LED without a bleaching gel allowed for a color change but was less effective than its use with HP, as observed in previous studies^3^ ^,^ ^4^. All groups showed a bleaching index consistent with their HP concentration, resulting in color changes at perceptible and clinically acceptable levels, as indicated in previous studies^21^ ^,^ ^22^. Those that received HP10% with the addition of CaPP, whether associated with LED or not, achieved a higher bleaching index. This is consistent with previous studies ^3^ ^,^ ^5^ ^,^ ^24^ in which LED enhanced the bleaching effect. Moreover, the addition of CaPP did not interfere with the bleaching efficacy of the groups to which it was added^23^.

It is important to recognize that while laboratory results provide valuable insights, caution must be exercised when applying them to clinical settings ^25^ ^,^ ^26^. This is particularly relevant for dental pulp, a highly specialized connective tissue known for its repair and regenerative capabilities. In our study, we used extracts from each experimental group directly on cultured cells, allowing us to assess cytotoxicity data and compare them over different analysis periods. However, the complexities of clinical environments must be taken into account. For example, the dentin of vital teeth contains tubules that house cytoplasmic extensions of odontoblasts, along with collagen and dentinal fluid, which is under constant exudation pressure^26^. These factors can significantly influence the diffusion of components released from dental materials into the pulp tissue. Thus, the impact of a dental material or clinical procedure on cells may vary, potentially causing less damage in vital teeth compared to laboratory conditions, where in vitro models attempt to simulate in vivo environments, as observed in our current study^25^.

The incorporation of calcium polyphosphate into the hydrogen peroxide gel showed excellent results in all applied tests (Figure 1,2,3,4,5), reducing cytotoxicity, increasing cell viability, lowering oxidative stress, and decreasing hydrogen peroxide diffusion into pulp cells. Additionally, it maintained bleaching efficacy, and the combination with violet LED further enhanced this outcome. In a clinical setting, this could imply a reduction in significant adverse effects, such as hypersensitivity, potentially leading to a safer and more comfortable treatment for patients. However, further studies are needed to confirm its clinical utility.

## Data Availability

The research data are available upon request.
